# ISO 9001:2015 standard implementation in clinical trial centers: An exploratory analysis of benefits and barriers in Italy

**DOI:** 10.1016/j.conctc.2023.101104

**Published:** 2023-03-11

**Authors:** V. Franchina, S. Stabile, R. Cenna, F. Mannozzi, I. Federici, S. Testoni, V. Sinno, C. Cagnazzo

**Affiliations:** a U.O.C. Oncologia Medica, A.O. Papardo Messina, Italy; b Niguarda Cancer Center, Dipartimento di Ematologia ed Oncologia Struttura Complessa Oncologia Falck Milano, Italy; c Unità di Ricerca e Sviluppo Clinico, S.C. Oncoematologia Pediatrica - AOU Città della Salute e della Scienza, Presidio Ospedaliero Infantile Regina Margherita, Torino, Italy; d Clinica di Ematologia, AOU Ospedali Riuniti di Ancona, Italy; e Unità di Biostatistica e Sperimentazioni Cliniche, IRCCS Istituto Romagnolo per lo Studio dei Tumori (IRST) “Dino Amadori”, Italy; f Pharma quality Europe, Reggello, Italy; g Gruppo Italiano Data Manager - Coordinatori di Ricerca Clinica, Meldola, Italy

**Keywords:** ISO, Good clinical practice, Quality, Quality standards, Benefits, Processes

## Abstract

**Background:**

In the last decade many clinical research centers in Italy have increasingly implemented and improved their quality standards and effectiveness of processes through the adoption of a quality management system also according to the certification ISO 9001:2015.

**Objective:**

The aim of this project is to evaluate expected benefits and barriers of ISO 9001 certification for a Clinical Trial Center.

**Material and methods:**

On April 2021, the Italian Group of Data Manager and Clinical Research Coordinator spread an anonymous online survey to healthcare professionals operating in clinical research and quality management systems at research sites.

**Results:**

Reported benefits of ISO oriented Quality Management System adoption include continual improvement and better-quality processes (73.3%), assuring corrective actions (63.6%), planning internal audits (60.2%) and risk management approach (60.7%). The most important barriers to QMS implementation are increased logistical and/or organizational activities (40.9%) and insufficient training on quality programs (29.5%).

**Conclusions:**

Implementing a quality management system represents a challenge for the Clinical Trial Center and helps to improve quality standards and risk management approach. The use of electronic tools is poor and could be increased in the future. Lastly, improvement of continuous QMS trainings should be necessary for updating professionals and optimizing activities within the Clinical Trial Center.

## 1 Introduction

In the last decade Clinical Trial Centers (CTC) in Italy have increasingly implemented and improved their quality standards through the adoption of quality management systems (QMS) defined not as a simple collection of procedures [1,2], but as part of the healthcare professionals’ services [3].

Generally, QMS ensures the organizations to measure, maintain and control quality through the adoption of plan-do-check-act (PDCA) processes to support continuous improvement of quality standards [4] also in healthcare organizations [5,6].

The importance of the QMS in clinical trials is also underlined in the last version of Good Clinical Practice (revision 2) [7] especially on the Sponsor's side with the request of high-quality standards obtained through quality assurance and a risk-based process, from trial design to conduct and termination [[8], [9], [10]].

Moreover, since 2015 in Italy the implementation of a QMS is also one of the minimum requirements imposed to research sites (both clinical departments and laboratories) for the conduct of phase 1 trials according to Italian Law [2,8].

In this scenario many CTC in Italy have been voluntarily registering to ISO 9001:2015 according to international standard, improving the efficiency and quality of healthcare services [11].

The ISO 9001:2015 is the current version of the ISO 9001 series for the Quality Management System. This standard, based on the High Level Structure (HLS) provides a process-oriented approach and establishes fundamental principles and requirements involved in the aspect of the quality [10,[12], [13], [14]].

**Fig. 1:**
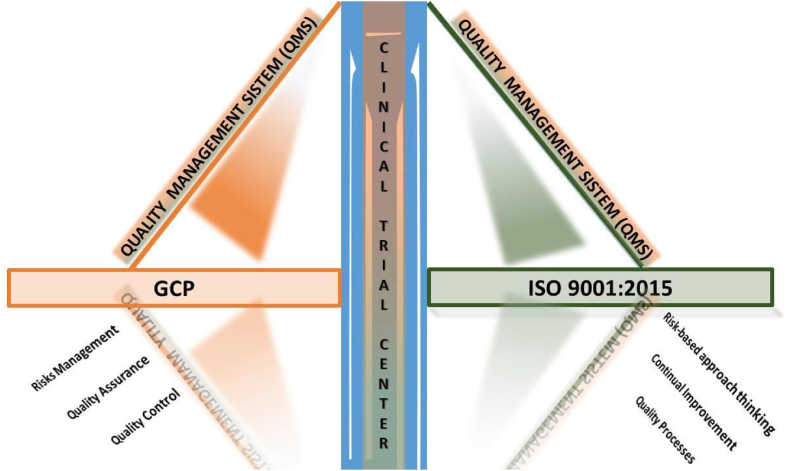
Quality management system in GCP and ISO 9001:2015 standards.

Although not overlapping with the GCP requirements, ISO 9001:2015 certification could help clinical trial sites in the adoption of a QMS model focused on a risk-based approach with a continual improvement driven by objective measurements [10,12] (Fig. 1).

The expected benefits [3] of a ISO 9001:2015 system adoption could lead to continuous improvement of the activities according to the requirements and the quality of the services provided [14,15]; identification and management of risks; patients expectations and satisfaction [3]; process performance; specific training of the clinical team; organizational and management aspects of the work team; SWOT (Strengths, Weaknesses, Opportunities, Threats) analysis of the internal and external context [12]; periodic management and review of documents produced required by the certification; internal audits; Risk management and assessment; implementation of corrective actions following detected non-conformities of the QMS.

Implementing a QMS in healthcare or in a hospital department, like a clinical research unit, is also helpful to define the strategic lines of organization, develop objectives, establish monitoring indicators, standardize the work of the Unit through procedures and protocols, increase safety at work through the use of lists of verification, initiate improvement actions to strengthen the deficiencies of the QMS itself, as well as learn the degree of satisfaction and needs of patients and the personnel who work in it [14].

The aim of this project is to evaluate expected benefits and barriers of ISO 9001 certification for the Italian Clinical Trial Center (CTC).

## 2 Methods and material

On April 2021, the Italian Group of Data Manager and Clinical Research Coordinator (GIDMcrc) spread an anonymous online survey to healthcare professionals operating in clinical research and quality management systems at clinical trial sites and Clinical Trial Centers. The questionnaire consisted of 15 multiple choice questions focused on QMS and ISO 9001:2015 certification for sites and CTC (Appendix 1). The survey instrument regarded mainly the characteristics of CTC (conducting clinical trial, presence of QMS); opportunities and advantages of certification ISO 9001:2015; expectations of respondents about the international standard.

The survey was designed by a pool of quality assurance managers and spread online through Google Forms® technology. Before online publication, the questionnaire was tested with a pilot test on a small group of clinical research coordinator and quality assurance managers, operating in the main Italian clinical research centers in the onco-hematological field. The final version of the survey incorporated the changes and additions resulting from the pilot phase.

The survey remained active for 5 months, from 20 April to September 30, 2021, then data were extracted and analyzed in October 2021.

## 3 Results

A total of 88 professionals have completed the survey (48.9% from north Italy, 39.8% south and 11.4% central). Most of respondents were clinical research coordinators (CRC) (64.8%, n = 57) followed by physicians (8%, n = 9), pharmacists, researchers (6.8%, n = 6) and research nurses (4.5%, n = 4) and Quality Assurance (QA) managers (3.3%, n = 3).

Oncologic CTC are the most represented facilities (61.4%, n = 54) followed by hematological (21,6%, n = 19) and neurological ones (5,7%, n = 5). Laboratories were poorly represented, with only 2 responders (2,2% overall).

Almost all respondents (93.2%, n = 82) routinely conduct phase II/III trials, 75% (n = 66) are involved in observational research and 61,4% (n = 54) conduct phase IV clinical trials. Only 33% (n = 29) of responders are also involved in phase 1 clinical trials.

A Good Clinical Practices’ QMS is present in 81.8% (n = 72) of cases and 76.3% (n = 55) of these are in compliance with ISO 9001:2015 standard requirements.

Furthermore, through an analysis limited to phase 1 structures 100% of these responders declares to have a QMS.

Not considering those not certified for phase 1 trials, the percentage of sites with a quality management system in place reaches 51%, 22% of which are in possession of a ISO 9001:2015 certification.

The most common electronic tools used are client/server or web-based software (55.7%, n = 49) and organizational databases (44.3%, n = 39).

**Fig. 2:**
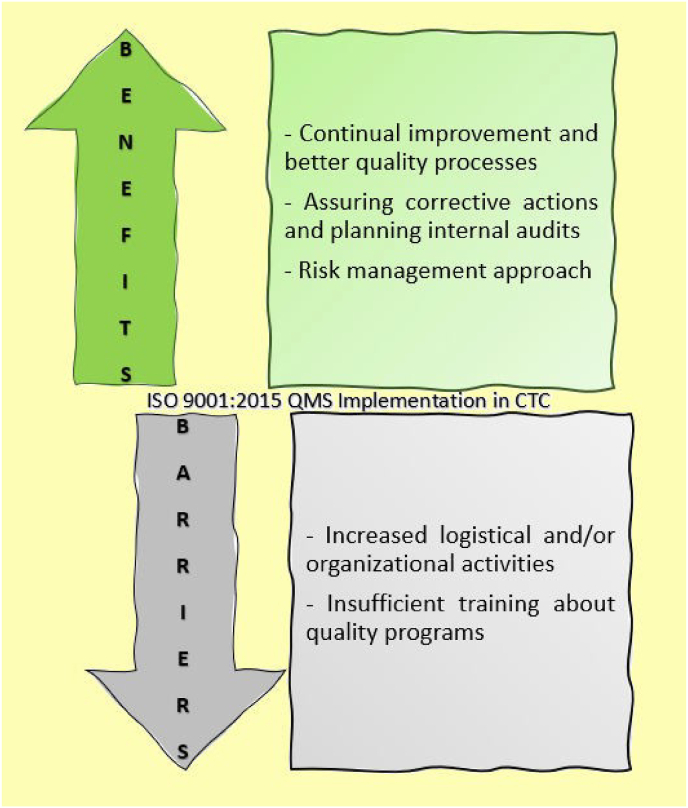
Benefits and Barriers of a ISO 9001:2015 system adoption in CTC.

Reported benefits of ISO oriented QMS adoption include: continual improvement and higher quality processes (73.3%, n = 65), assuring corrective actions (63.6%, n = 56), planning internal audits (60.2%, n = 53) and risk management approach (58%, n = 51). The most important perceived barriers to QMS implementation are: increased logistical and/or organizational activities (40.9%, n = 36) and insufficient training on quality programs (29.5%, n = 26) (Fig. 2). Particularly, 45.5% (n = 40) of professionals have not performed at least one QMS training in the last year and 54.5% (n = 48) of clinical research centers have never organized specific training on QMS (Appendix 2).

## 4 Discussion

The results obtained represent a well photography of the Italian clinical trial units' scenario despite some study's limits due to the intrinsic nature of an anonymized survey (possible biases in responses based on responders' expertise or impossibility to characterized well the sample due to anonymization). Despite the limitation described, the data obtained highlight the importance of implementing, in centers that conduct clinical research activities in Italy, a quality management system (QMS) in compliance with the ISO 9001: 2015 standard in order to ensure continuous monitoring of the activities carried out, the analysis of results, risk analysis and management of deviations leading to continuous improvement. Based on swot analysis, the main strong point therefore is represented by the ever-increasing importance of structuring research sites into increasingly structured and well-defined entities from an organizational, administrative and logistical point of view, bringing greater attention to those processes that characterize the entire quality “management system” in support of the activities carried out in clinical research facilities.

These principles are meant to integrate, not replace, the essential ones dictated by Good Clinical Practice (GCP). The combination of the two standards represents an added value and an opportunity for a CTC to offer services as well as innovative and high-quality processes.

As pointed out by Kunga et al. [16], several studies highlight the need to distinguish the two standards which, despite with similar purposes, have different objectives.

GCPs are certainly the mandatory standard of excellence that every clinical trial must comply with in terms of human subject protection and data integrity.

ISO 9001: 2015, on the other hand, represent the voluntary and active choice by a structure to adopt a “system” standard in terms of quality and systematic management of processes, resources and services based on study design and development, data processing, measurement, analysis and improvement, not limited to a document system.

Through these aspects, both international standards, albeit with different principles, contribute to the achievement of reliable results, in order to guarantee the excellence in terms of quality control, risk management, continuous improvement of the processes carried out, patient involvement in healthcare through the adoption and development of patient's satisfactions activities.

The results of our work, however, have highlighted the difficulties that some CTCs face in undertaking the process of ISO 9001: 2015 certification for reasons such as:

- logistics: high contractual costs related to the certification and annual audits to confirm certification
- organizational: further implementation of SOPs dedicated to the standard and related document management, planning periodic internal audits relating to the standard
- training: activation training program specifically focused on the achievement of the objectives set out by the various points of the standard.

It also appears that a QMS GCP is present in all clinical centers that conduct phase 1 trials, which are more accustomed to working within a quality management system.

It is therefore necessary, in order to maintain the SGQ in line with ISO guideline and, more generally, with GCP, to seek a continuous improvement of activities performed according to requirements and high quality services guaranteed through the adoption of innovative and validated electronic tools to ensure an adequate data and risk management aimed at increasingly reliable data. These aspects, as survey responses suggest, are currently still identified as weak points.

Ultimately, the threats perceived around the implementation of the SGQ are clearly linked to the increase in logistical and organizational activities, inconsistent training regarding quality programs and high costs that sites would have to sustain in order to obtain the certification.

The lack of specific training on quality management and quality assurance of the personnel involved in clinical research certainly represents an important issue detected in this survey.

## 5 Conclusion

This project has an exploratory purpose and is not intended as an accurate description of the Italian landscape due to the failure to identify an initial sample aimed at a more heterogeneous analysis. However, it is clear that the adoption of a quality management system by the centers that conduct clinical trials in Italy does not yet represent a widespread requirement in all centers in Italy, although common in those sites conducting phase 1 trials. This is most likely due to the publication of the AIFA Determination 809/2015, which requires the Italian phase I centers to adopt a QMS.

The implementation of a quality management system, that would help improve quality standards through the application of a risk management approach to the various ongoing processes, represents an important challenge for a clinical research center. In this perspective, it becomes necessary to adopt increasingly innovative and validated electronic tools in order to guarantee correct data management and risk management in order to obtain increasingly reliable results. Furthermore, continuous training of the QMS is essential to keep professionals up to date and optimize their activities at the CTC. Finally, the combination of the two standards represents an added value and an opportunity for a CTC to offer qualitative and innovative services and processes to the patient who always remains the undisputed protagonist of his clinical care path.

## Declaration of competing interest

The authors declare that they have no known competing financial interests or personal relationships that could have appeared to influence the work reported in this paper.
