# Supplementary material for: ISO 9001:2015 standard implementation in clinical trial centers: An exploratory analysis of benefits and barriers in Italy
Source: Contemp Clin Trials Commun. 2023 Mar 11;33:101104. doi: 10.1016/j.conctc.2023.101104 (PMC10313875; doi:10.1016/j.conctc.2023.101104)
Supplement: Multimedia component 1 [file mmc1.doc]

# Appendix 1

**SURVEY**

| **1. Region sites in which you operate** | - North Italy - South Italy - Central Italy |
| --- | --- |
| **2. What role do you play in your hospital?** | - Physician - Clinical Research Coordinator (CRC) - Research Nurse - Pharmacist - Researcher - Quality assurance - Other |
| **3. In which field do you carry out your clinical research activity?** | (*Open-ended question)* |
| **4. What types of clinical studies are carried out at your facility?** | - Phase I - Phase II/III - Phase IV - Observational Studies - No studies are conducted |
| **5. Does your facility has a quality management system?** | - Yes - No - I don’t Know |
| **6. If YES, did your QMS certified ISO 9001:2015?** | - Yes - No |
| **7. If YES, how long have you been ISO 9001:2015 certified?** | - From one year - First surveillance audit - Second surveillance audit - Re-certification |
| **8. If there is NO Quality Management System, does the facility intend to activate an ISO 9001:2015 certification process?** | - Yes, in the next months - No activities for certification - No a cause of high cost |
| 1. **What IT tools are used in your center in order to guarantee your quality management system?**   (Multiple choice questions) | - Electronic/web document management - Client/server or web based software for corporate shared use - Organizational databases - Intranet workflow - No specific instruments are adopted |
| **10. In your opinion, what are the expectations of a clinical research center that decides to obtain ISO 9001:2015 certification?**  (Multiple choice questions) | - More efficient operating process - Better risk management - Better use of resources - Greater visibility - I don’t know |
| **11. In your opinion, what are the benefits for a center to obtain ISO 9001:2015 certification?** | - Periodic management reviews and internal audits - Continuous improvement through process optimization and productivity improvement |

| (Multiple choice questions) | - Standardized and formal corrective action procedures - Risk management - Control activities and processes - Conduct frequent user feedback surveys |
| --- | --- |
| **12. What do you think are the main barriers for a clinical center to implement ISO9001 certification?**  (Multiple choice questions) | - Increase in bureaucratic activities - Logistical and organizational barriers - Contractual costs - Lack of proper training - We find no other critical issues - Other |
| **13. Have your hospital organized specific courses on quality management systems?** | - Yes, in the context of clinical trials - Yes, also in reference to ISO systems - No |
| **14. Have you received specific training on quality management systems in the last year?** | - Yes, in the context of clinical trials - Yes also in reference to ISO systems - No |
| **15. If you have training in the field of quality management systems, what kind?**  (Multiple choice questions) | - Webinar - Advanced Courses - Virtual Meeting - Master’ degree - Other |

| **Appendix 2 SURVEY RESULTS**  **No. 88 respondents** |  |
| --- | --- |
| 1. **Region sites in which you operate**  - 48,9% North Italy - 39,8 % South Italy - 11,4% Central Italy | **9. What IT tools are used in your center in order to guarantee your quality management system?**   - 55,7% Electronic/web document management - 55,7% Client/server or web based software for corporate shared use - 44,3% Organizational databases - 19,3% Intranet workflow - 13,6% No specific instruments are adopted |
| 1. **What role do you play in your hospital?**  - 8% Physician - 64,8% Clinical Research Coordinator (CRC) - 4,5% Research Nurse - 6,8% Pharmacist - 4,5% Researcher - 3,3% Quality assurance - 2,2% Other | **10. In your opinion, what are the expectations of a clinical research center that decides to obtain ISO 9001:2015 certification?**   - 83% More efficient operating process - 65,9% Better risk management - 56,8% Better use of resources - 34,1% Greater visibility - 2,3% I don’t know |
| 1. **In which field do you carry out your clinical research activity?**   **(*Open-ended question)***   - 61.4% Oncology - 21,6% Ematology - 5,7% Neurology - 2,3% Cardiology - 2,2% Laboratory | **11. In your opinion, what are the benefits for a center to obtain ISO 9001:2015 certification?**   - 60,2% Periodic management reviews and internal audits - 73,3% Continuous improvement through process optimization and productivity improvement - 63,6% Standardized and formal corrective action procedures - 58% Risk management - 58% Control activities and processes - 19,3% Conduct frequent user feedback surveys |
| 1. **What types of clinical studies are carried out at your facility?**  - 33% Phase I - 93,2% Phase II/III - 61,4% Phase IV - 75% Observational Studies | **12. What do you think are the main barriers for a clinical center to implement ISO9001 certification?**   - 40,9% Increase in bureaucratic activities - 39,8% Logistical and organizational barriers - Contractual costs - Lack of proper training - We find no other critical issues - Other |
| 1. **Does your facility has a quality management system?**  - 81,8%Yes - 12,5% No - 5,7% I don’t Know | **13. Have your hospital organized specific courses on quality management systems?**   - 22,7% Yes, in the context of clinical trials - 22,7% Yes, also in reference to ISO systems - 54,5% No |
| 1. **If YES, did your QMS certified ISO 9001:2015?**  - 76,3% Yes - 23,7% No | **14. Have you received specific training on quality management systems in the last year?**   - 34,1% Yes, in the context of clinical trials - 20,5% Yes also in reference to ISO systems - 45,5% No |
| 1. **If YES, how long have you been ISO 9001:2015 certified?**  - 6,6% From one year - 16,7% First surveillance audit - 15% Second surveillance audit - 48,3% Re-certification | **15. If you have training in the field of quality management systems, what kind?**   - 67,3% Webinar - 24,4% Advanced Courses - 18,4% Virtual Meeting - 10,2% Master’ degree - 2% Other |
| 1. **If there is NO Quality Management System, does the facility intend to activate an ISO 9001:2015 certification process?**  - 18,5% Yes, in the next months - 3,7% No activities for certification - 3,7% No a cause of high cost |  |
